# Effects of psychological interventions on competitive anxiety and athletic performance in golfers: a systematic review and meta-analysis

**DOI:** 10.1186/s13102-026-01888-2

**Published:** 2026-08-27

**Authors:** Longhui Li, Zilong Zhao, Xuewen Li, Yang Yi

**Affiliations:** 1 Capital University of Physical Education and Sports, Beijing, China; 2 Hebei Sport University, Shijiazhuang, 050041 China

**Keywords:** Athletic performance, Competitive anxiety, Golf, Meta-analysis, Psychological intervention

## Abstract

**Background:**

Competitive anxiety is common in golf and can negatively affect athletic performance. Psychological interventions are widely used to manage competitive anxiety and enhance performance; however, evidence specific to golfers has not yet been systematically synthesized. This study aims to evaluate the effects of psychological interventions on competitive anxiety and athletic performance in golfers. In addition, subgroup analyses were conducted to explore potential sources of variation in intervention effects.

**Methods:**

A systematic search of Cochrane, Embase, PubMed, Scopus, and Web of Science identified randomized and non-randomized controlled trials evaluating psychological interventions to reduce competitive anxiety or improve athletic performance in golfers. Risk of bias was assessed using the revised Cochrane risk-of-bias tool for randomized trials and the Risk Of Bias In Non-randomized Studies of Interventions tool. Certainty of evidence was evaluated using the Grading of Recommendations Assessment, Development and Evaluation approach. Meta-analyses were performed using Stata 18.0.

**Results:**

A total of 15 studies involving 606 participants were included. Nine studies were rated as low risk of bias, four as having some concerns, and two as moderate risk of bias. Psychological interventions were associated with reductions in competitive anxiety (SMD = − 0.29, 95% CI: −0.57 to − 0.02) and improvements in athletic performance (SMD = 0.70, 95% CI: 0.28 to 1.12). Substantial heterogeneity was observed for athletic performance outcomes (I² = 80.3%). Exploratory subgroup analyses suggested that effects may be more evident among male and amateur golfers and in studies using interventions delivered two to three times per week for sessions lasting up to 30 min.

**Conclusions:**

Psychological interventions were associated with small reductions in competitive anxiety and moderate improvements in athletic performance in golfers. However, the certainty of the evidence was low, and substantial heterogeneity, limited evidence for subgroup analyses, and potential publication bias should be considered when interpreting the findings. Future research should prioritize high-quality randomized controlled trials with more balanced representation across gender and competitive level, and further examine specific dimensions of competitive anxiety as well as emerging approaches such as mindfulness-based interventions and technology-assisted methods including virtual reality and biofeedback.

**Trial registration:**

PROSPERO: CRD420251025181.

**Supplementary Information:**

The online version contains supplementary material available at https://doi.org/10.1186/s13102-026-01888-2.

## Introduction

Competitive anxiety is a widespread psychological challenge in sport and refers to the emotional and physiological responses athletes experience in competitive situations. It is commonly conceptualized as either a stable predisposition (trait anxiety) or a situational reaction to competitive stress (state anxiety), expressed through both cognitive and bodily symptoms [1, 2]. These responses may include worry, negative self-evaluation, fear of failure, elevated heart rate, muscle tension, and other stress-related reactions [1]. According to the multidimensional anxiety theory proposed by Martens et al. (1990), competitive anxiety comprises two primary components: cognitive anxiety and somatic anxiety [3]. Cognitive anxiety involves concerns, intrusive thoughts, and performance-related worries, whereas somatic anxiety reflects physical arousal such as increased heart rate, rapid breathing, sweating, and muscular tension [3]. When elevated, these forms of anxiety can disrupt attentional control, reduce processing efficiency, and negatively affect athletic performance, while also increasing susceptibility to injury [1, 4, 5].

Subsequent research has refined this framework. Hierarchical models conceptualize competitive anxiety as comprising cognitive anxiety, somatic anxiety, and self-confidence as distinct yet interrelated dimensions, emphasizing the functional role of self-confidence in performance contexts [6]. More recent perspectives adopt dynamic, process-oriented approaches, highlighting the role of psychological resources such as resilience and self-regulation in shaping anxiety responses [7, 8], as well as the influence of basic psychological needs (e.g., competence and autonomy) within self-determination theory [9]. Competitive anxiety is also increasingly understood within a broader system involving cognitive appraisal, coping processes, and contextual factors such as social evaluation [10]. Measurement-based models have further extended traditional frameworks by incorporating attentional constructs (e.g., concentration), underscoring the interaction between anxiety and attentional control in sport performance [11].

Golf appears particularly vulnerable to the effects of competitive anxiety due to its technical precision, self-paced structure, and high cognitive demands. Empirical evidence indicates that anxiety levels increase during critical moments, especially during short putts (≤ 1.5 m), where the perceived consequences of error are high [12, 13]. Performance pressure in golf is further amplified by the social environment in which competition occurs. Golfers, regardless of skill level, perform under close observation from coaches, competitors, and spectators, making them especially sensitive to evaluation and judgment [14, 15]. Recent research highlights the situational dynamics of pressure in golf. For instance, the “first-hole effect” reflects elevated anxiety and performance variability at the onset of competition, likely driven by anticipatory stress and adjustment demands [16]. Pressure also varies across the course of competition and may intensify in situations perceived as more demanding, such as performance-critical moments, where cognitive load and fear of failure are elevated [17, 18]. Under these conditions, concerns about negative evaluation, fear of disappointing others, and heightened self-awareness may increase social anxiety and impair attentional control and motor execution [15, 19, 20]. In particular, anxiety-related shifts toward self-focused processing can disrupt the automaticity of well-learned motor skills, a mechanism that is especially detrimental in precision-based tasks such as golf putting [12, 16]. As a result, these psychological demands may compromise movement coordination, contribute to performance breakdowns, and are closely associated with maladaptive phenomena such as the “yips” [12, 21, 22]. Accordingly, effective management of competitive anxiety represents a key target for psychological interventions aimed at optimizing performance in golfers.

Psychological interventions are widely recognized as effective strategies for regulating competitive anxiety in athletes. Recent meta-analytic evidence indicates that these approaches produce moderate-to-large reductions in anxiety across controlled trials [23]. Complementary findings from a systematic review and Bayesian network meta-analysis suggest that effectiveness varies across intervention types, with consistent support for both cognitive-behavioral approaches and mindfulness- and acceptance-based interventions [24]. This evidence provides a strong empirical basis for their application in sport-specific contexts, including golf [25]. Among these approaches, imagery is one of the most commonly used techniques, enabling golfers to mentally rehearse body posture, grip, and shot execution, thereby enhancing skill familiarity and facilitating better regulation of arousal and anxiety [26–28]. Other interventions, including relaxation techniques [29], mindfulness [30], psychological skills training [31], and cognitive behavioral therapy (CBT) [20, 32], have also demonstrated effectiveness in reducing anxiety and improving performance in golfers. Beyond anxiety regulation, psychological interventions may further support skill acquisition and the refinement of performance under competitive conditions [22, 33].

Although growing evidence supports the effectiveness of psychological interventions in reducing competitive anxiety and enhancing performance in athletes, no systematic review and meta-analysis has specifically synthesized their effects on competitive anxiety and athletic performance in golfers. Furthermore, it remains unclear how factors such as intervention type, duration, and athletes’ competitive level may influence these outcomes within golf contexts. To address this gap, the present study provides the first golf-specific systematic review and meta-analysis of the effects of psychological interventions on competitive anxiety and athletic performance in golfers. In addition, subgroup analyses were conducted to examine how intervention characteristics and participant demographics may shape intervention effectiveness. By synthesizing the available evidence in this specific sporting context, this study aims to inform future research and provide evidence-based guidance for practitioners seeking to optimize psychological preparation strategies in golf. Accordingly, we expected that psychological interventions would be associated with reductions in competitive anxiety and improvements in athletic performance among golfers. Given the heterogeneity of interventions and participant characteristics, we further anticipated that intervention effectiveness might vary across different intervention and demographic subgroups.

## Materials and methods

### Protocol and registration

This review followed the Preferred Reporting Items for Systematic Reviews and Meta-Analyses (PRISMA) guidelines [34], with the checklist provided in Supplementary Material 1. It has been registered with the International Prospective Register of Systematic Reviews (PROSPERO) under registration number CRD420251025181, which is accessible at https://www.crd.york.ac.uk/prospero/.

### Search strategy and selection criteria

A comprehensive systematic search was conducted across Cochrane, Embase, PubMed, Scopus, and Web of Science databases, covering the period from inception to March 2025. Additional citations were sourced from Google Scholar and relevant review articles. The search was conducted in accordance with the guidelines of the Cochrane Handbook for Systematic Reviews [35]. The full search strategy is detailed in Table S1. Two researchers (LLH and YY) independently conducted the search, and any disagreements were resolved by consulting a third researcher, LXW.

Studies were included if they met the following PICOS-based criteria: (1) Population: Golf athletes of any level; non-golf athletes were excluded. (2) Interventions: Psychological interventions such as cognitive-behavioral therapy, psychological skills training, imagery, mindfulness, or relaxation. Non-psychological interventions were excluded. (3) Control group: No intervention, wait-list controls, or regular training. (4) Outcomes: Measures of competitive anxiety (e.g., Competitive State Anxiety Inventory, Sport Anxiety Scale, State-Trait Anxiety Inventory, Sport Competitive Anxiety Test) and direct indicators of golf performance (e.g., 18-hole stroke count, putting performance, short game performance). (5) Study design: Controlled trials, including randomized and non-randomized designs, with at least one control group. Only English-language publications were considered.

### Study selection

All identified records were managed using EndNote X9. After duplicate removal, the screening process was conducted independently by two researchers, LLH and YY. The investigators initially evaluated the eligibility of the remaining studies by reviewing the title and abstract. Subsequently, full-text reports were reviewed to determine their suitability for inclusion criteria. At each stage, both researchers need to agree, and any disagreements are resolved by reaching consensus with the third researcher (LXW). Inter-rater reliability at the full-text stage was assessed using the kappa statistic.

### Data extraction

Data were independently extracted by two reviewers (LLH and YY) and cross-checked for accuracy, with discrepancies resolved by a third reviewer (LXW). Extracted data included: author, year of publication, country, participant characteristics (age, gender, sample size, competitive level), intervention details (type, length, frequency, duration), and measurement tools. For the purposes of this review, golfers were classified into four levels of expertise: elite (national-level or top junior competitors), semi-elite (regional or provincial competitors), amateur (individuals with limited competitive experience), and novice (beginners with no formal competition experience). This classification framework was predefined prior to data extraction and applied consistently across studies. Classification was based on the information reported in the original studies, including participants’ competitive level and descriptive characteristics; when explicit categorization was not provided, levels were determined based on the available contextual information.

### Study risk of bias assessment

Two reviewers (LLH and YY) independently assessed the risk of bias of the included studies, with disagreements resolved through discussion and, when necessary, consultation with a third reviewer (LXW). The 13 randomized controlled trials were evaluated using the revised Cochrane Risk of Bias tool for randomized trials (RoB 2), which assesses bias arising from the randomization process, deviations from intended interventions, missing outcome data, measurement of the outcome, and selection of the reported result. The two non-randomized controlled studies were assessed using the updated Risk Of Bias In Non-randomised Studies of Interventions tool (ROBINS-I V2), which evaluates bias across six domains: confounding, classification of interventions, selection of participants into the study (or into the analysis), missing data, measurement of outcomes, and selection of the reported result [36].

### Data preparation

All outcome measures were standardized prior to analysis. When scales differed in directionality (e.g., higher scores indicating either improved or reduced performance), means were multiplied by − 1 to ensure consistency [37]. For studies with multiple intervention groups (e.g., by gender or age), pooled means and standard deviations were calculated. If several instruments assessed the same outcome, the primary measure was prioritized; if no primary measure was reported, results across scales were averaged. Final outcome selection was determined by consensus among three authors (LLH, YY, LXW), guided by theoretical considerations.

### Meta-analysis

The meta-analysis was conducted using Stata 18.0, a statistical software package with comprehensive functions for synthesizing data across study designs [38]. Statistical heterogeneity was quantified using the I² statistic. Given the expected clinical and methodological heterogeneity among the included studies, random-effects models were applied for all analyses to provide more conservative estimates of the pooled effects [39]. For outcomes with substantial heterogeneity, meta-regression analyses were performed to explore potential sources. To account for differences in outcomes and measurement scales, standardized mean differences (SMD) with 95% confidence intervals (CI) were calculated. Exploratory subgroup analyses were performed according to categorical variables, including participant characteristics (e.g., gender and competitive level), intervention features (e.g., type, duration, frequency, and session length), and outcome measures, to examine potential patterns in intervention effects across study characteristics. Publication bias was assessed using funnel plots and Egger’s test, and, when appropriate, further examined using the trim-and-fill method. Sensitivity analyses were also conducted to assess the robustness of the findings by excluding studies with small sample sizes (*n* < 20) and non-randomized controlled trials [40, 41].

### Quality of evidence

The certainty of evidence was evaluated using the Grading of Recommendations Assessment, Development, and Evaluation (GRADE) approach [42]. Evidence quality was assessed across five domains—study limitations, consistency, directness, precision, and publication bias—and rated as high, moderate, low, or very low [42].

## Result

### Literature screening process and results

**Fig. 1:**
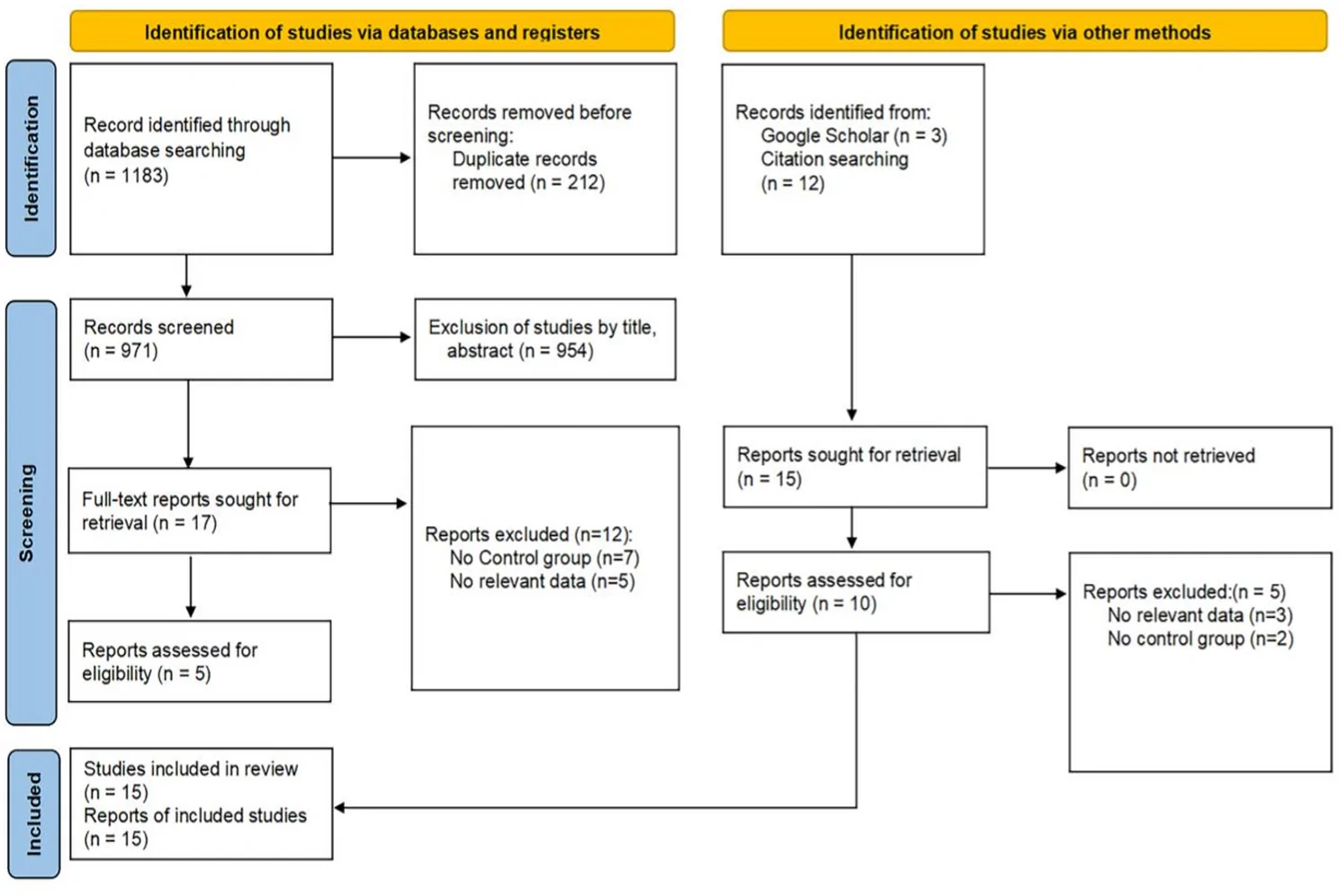
PRISMA flow chart

A total of 1,183 records were identified through database searches, with an additional 15 studies retrieved through manual screening. After removing duplicates in EndNote, 971 records remained. Title and abstract screening reduced this number to 32 potentially eligible studies. Following full-text review, 15 studies met the inclusion criteria and were included in the meta-analysis. Inter-rater agreement during full-text screening was excellent, with a kappa statistic of 1. Figure 1 presents the study selection process, and Table S2 provides a list of excluded studies along with reasons for exclusion.

### Study characteristics

Table S3 presents the characteristics of the 15 studies included in this meta-analysis, encompassing 606 participants in total. The studies were published between 1983 and 2022, with sample sizes ranging from 14 to 75 participants (mean = 40). The average participant age was 26.15 years, and female participants represented 26.7% of the total sample. Seven studies included both male and female participants, seven focused exclusively on male participants, and one included only female participants. Geographically, most studies were conducted in the United States (*n* = 10), followed by the United Kingdom (*n* = 2), China (*n* = 1), Malaysia (*n* = 1), and South Africa (*n* = 1). With respect to competitive level, 11 studies involved amateur golfers, three examined novices, and one included semi-elite athletes.

Two studies employed multi-arm designs, comparing interventions such as imaginal exposure with CBT, or imagery with relaxation. Among single-arm studies, the most frequently applied intervention was imagery (*n* = 4). Other approaches included goal setting (*n* = 2), psychological skills training (*n* = 2), relaxation (*n* = 2), CBT (*n* = 1), mindfulness (*n* = 1), and Mindfulness-Acceptance-Commitment (*n* = 1). Intervention duration ranged from 1 to 54 weeks, with session frequencies of 1–4 times per week and lengths between 10 and 90 min. The most commonly used measure of competitive anxiety was the Competitive State Anxiety Inventory-2 (CSAI-2, *n* = 3). Other tools included the revised CSAI-2R (*n* = 1), Modified CSAI-2 (*n* = 1), Sport Anxiety Scale-2 (SAS-2, *n* = 1), Sport Competition Anxiety Test (SCAT, *n* = 1), and State-Trait Anxiety Inventory (STAI, *n* = 1). In terms of performance outcomes, seven studies reported putting performance, two assessed 18-hole scores, and one examined short game performance. Regarding study design, thirteen studies were randomized controlled trials, while two were non-randomized controlled studies.

### Risk of bias assessment

Among the 13 randomized controlled trials, 9 were judged to be at low overall risk of bias, while 4 were judged as raising some concerns. Detailed ROB 2 assessments are provided in Table S4. Across the individual domains, bias arising from the randomization process was judged to be at low risk in 11 studies and as raising some concerns in 2 studies. Bias due to deviations from intended interventions was judged to be at low risk in 11 studies, with 2 studies raising some concerns. Bias due to missing outcome data was rated as low risk in 12 studies and as raising some concerns in 1 study. All RCTs were judged to be at low risk of bias for the measurement of the outcome and the selection of the reported result.

The two non-randomized controlled studies were assessed using the ROBINS-I tool. Both studies were judged to be at an overall moderate risk of bias. At the domain level, both studies were rated as having a moderate risk of bias due to confounding and in the selection of participants into the study. All remaining domains, including the classification of interventions, missing data, measurement of outcomes, and selection of the reported result, were judged to be at low risk of bias. Detailed ROBINS-I assessments are presented in Table S5.

### Results of meta-analysis

**Fig. 2:**
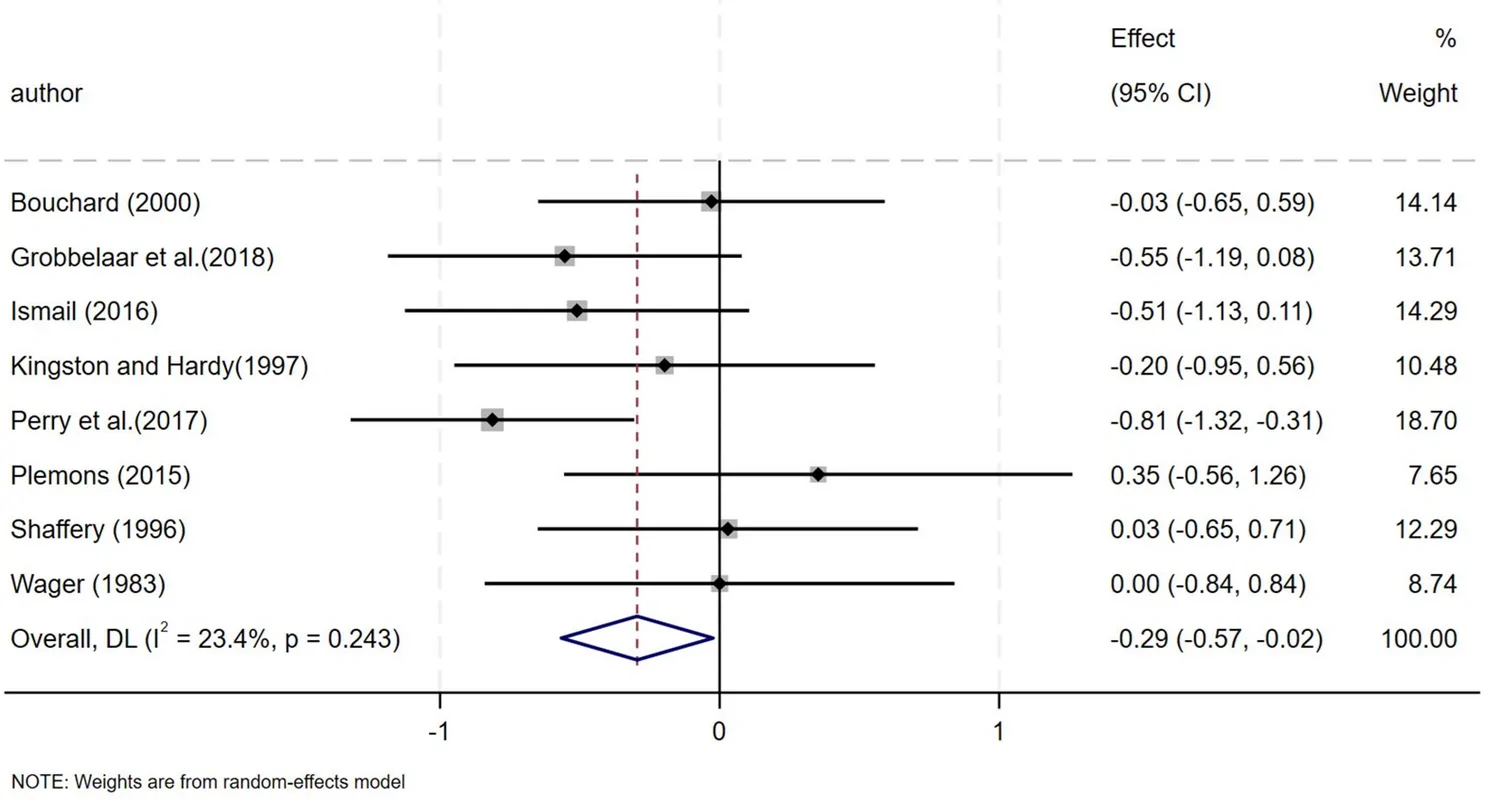
Forest plot of competitive anxiety outcomes

**Fig. 3:**
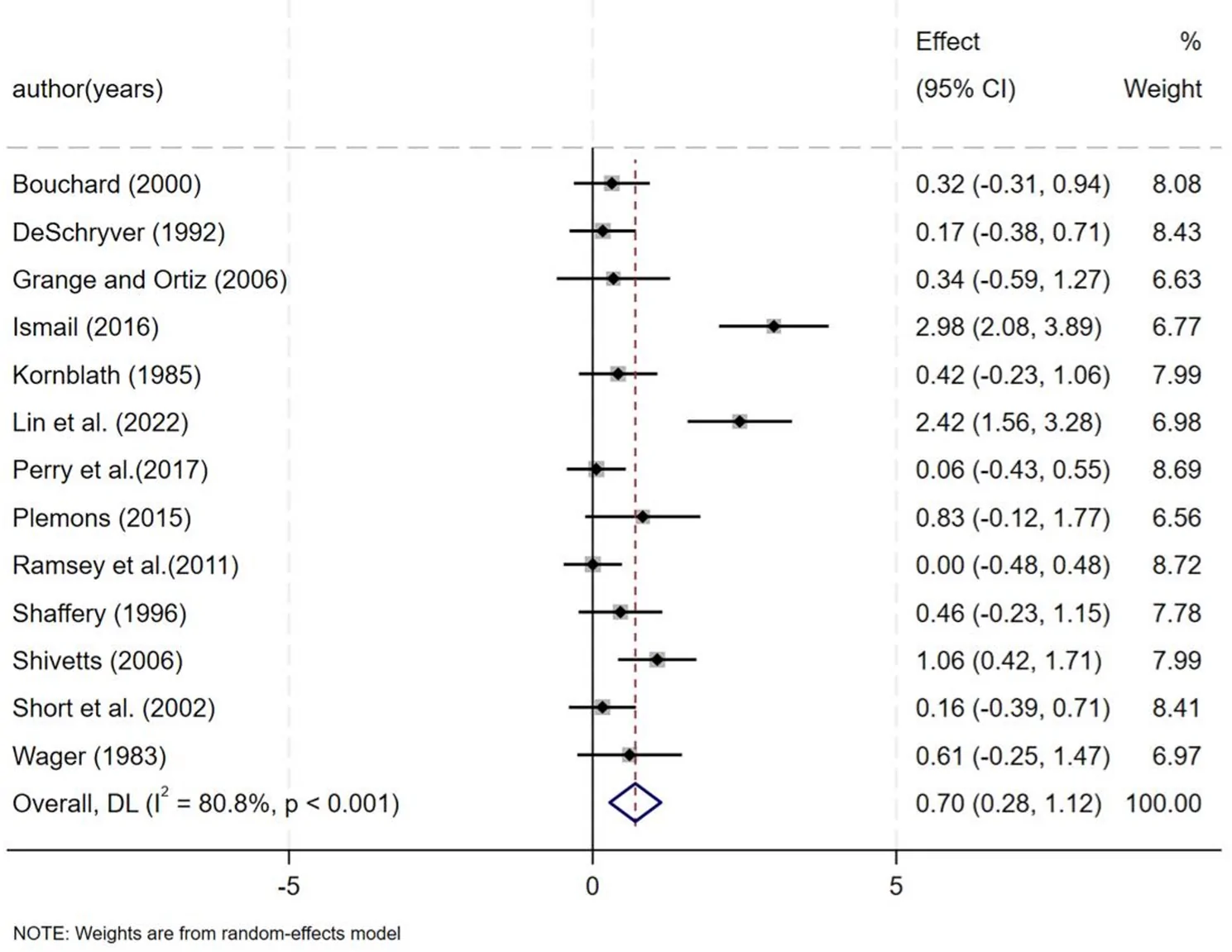
Forest plot of athletic performance outcomes

Meta-analyses were conducted for competitive anxiety and athletic performance outcomes using random-effects models. For competitive anxiety, low heterogeneity was observed (I² = 23.4%). As shown in Fig. 2, psychological interventions were associated with a significant reduction in competitive anxiety compared with control conditions (SMD = − 0.29, 95% CI: −0.57 to − 0.02, *p* < 0.05). In contrast, athletic performance outcomes exhibited substantial heterogeneity (I² = 80.3%). Figure 3 indicates that psychological interventions significantly improved athletic performance (SMD = 0.70, 95% CI: 0.28 to 1.12, *p* < 0.01). However, given the substantial heterogeneity across studies, this pooled estimate should be interpreted with caution. Meta-regression analyses were conducted to explore potential sources of heterogeneity, including participant characteristics (age, gender, region, and athlete level) and intervention characteristics (duration, frequency, session length, and type). Nevertheless, exploratory subgroup analyses suggested potential differences in effect sizes across certain study characteristics, particularly intervention frequency, although these findings should be interpreted cautiously. (Table 2 and Table S6).

#### Results of subgroup analysis

##### Athlete level and sex

Exploratory subgroup analyses were conducted according to athlete level and gender (Table 1). Psychological interventions were associated with improvements in competitive anxiety and athletic performance in both male and female golfers, with relatively larger effect sizes observed among male golfers (competitive anxiety: SMD = − 0.36, 95% CI: −0.71 to − 0.10; performance: SMD = 1.14, 95% CI: 0.28 to 1.99). However, these findings should be interpreted cautiously, as female participants were underrepresented in the included studies and only one female-only study contributed to the athletic performance analyses, limiting the ability to draw firm conclusions regarding sex differences. Across competitive levels, interventions appeared to be beneficial for golfers of all levels, although the magnitude of the effects varied. Effect sizes tended to be larger among amateur golfers (competitive anxiety: SMD = − 0.36, 95% CI: −0.63 to − 0.10; performance: SMD = 0.69, 95% CI: 0.25 to 1.14). These findings should be interpreted cautiously given the exploratory nature of the analyses and the limited number of studies in some subgroups.

**Table 1:** Subgroup analysis based on athlete level and gender

Outcome	Covariates	*N*	df	*p*-value	I^2^	Effect Size	95% CI	
Competitive Anxiety	Male	5	4	0.19	34.40%	-0.36	-0.71	-0.10
	Mixed Gender	3	2	0.25	29.40%	-0.14	-0.66	0.38
	Amateur	7	6	0.31	15.20%	-0.36	-0.63	-0.10
	Novice	1	0	NA	NA	-0.37	-0.54	1.28
Athletic Performance	Male	5	4	0.00	83.40%	1.14	0.28	1.99
	Female	1	0	NA	NA	0.34	-0.59	1.27
	Mixed Gender	7	6	0.00	77.50%	0.46	-0.01	0.93
	Amateur	10	9	0.00	74.50%	0.69	0.25	1.14
	Novice	3	2	0.00	92.20%	0.77	-0.13	1.97

*N *Number of studies, *NA *Not available

##### Intervention type, length, frequency, duration

Exploratory subgroup analyses were conducted according to intervention type, duration, frequency, and session length (Table 2; Figures S1 and S2). Effect sizes varied across intervention characteristics. Mindfulness-based interventions tended to be associated with larger reductions in competitive anxiety (SMD = − 0.82, 95% CI: −1.33 to − 0.32), whereas imagery interventions showed relatively larger improvements in athletic performance (SMD = 1.26, 95% CI: 0.11 to 2.41). Programs lasting longer than six weeks tended to be associated with larger reductions in competitive anxiety (SMD = − 0.82, 95% CI: −1.33 to − 0.32), whereas interventions lasting 3–6 weeks showed relatively larger improvements in athletic performance (SMD = 1.08, 95% CI: 0.43 to 1.74). Interventions delivered twice weekly tended to show larger effect sizes for both competitive anxiety (SMD = − 0.43, 95% CI: −1.26 to 0.41) and athletic performance (SMD = 2.69, 95% CI: 2.07 to 3.31), whereas lower or higher frequencies were associated with comparatively smaller effects. Similarly, session durations of up to 30 min tended to show larger effect sizes for competitive anxiety (SMD = − 0.30, 95% CI: −0.99 to 0.39) and athletic performance (SMD = 0.95, 95% CI: 0.06 to 1.84), whereas longer sessions were associated with smaller effects. Given that no statistically significant moderators were identified in the meta-regression analyses, these subgroup findings should be considered exploratory and interpreted with caution.

**Table 2:** Subgroup analysis based on length, frequency, and duration

Outcome	Covariates		*N*	df	*p*-value	I^2^	Effect Size	95% CI	
Competitive Anxiety	Length (weeks)	<3	3	2	0.25	28.20%	-0.13	-0.61	0.34
		3–6	4	3	0.59	0.00%	-0.22	-0.57	0.14
		> 6	1	0	NA	NA	-0.82	-1.33	-0.32
	Frequency (per week)	1	4	3	0.37	5.30%	-0.13	-0.50	0.24
		2	2	1	0.05	74.20%	-0.43	-1.26	0.41
		3	1	0	NA	NA	-0.20	-0.95	0.55
		5	1	0	NA	NA	-0.42	-1.14	0.10
	Duration (minutes)	≤ 30	3	2	0.06	64.40%	-0.30	-0.99	0.39
		30–60	1	0	NA	NA	0.00	-0.84	0.84
		60	4	3	0.43	0.00%	-0.28	-0.60	0.04
Athletic Performance	Length (weeks)	<3	4	3	0.88	0%	0.11	-0.15	0.38
		3–6	8	7	0.00	84%	1.08	0.43	1.74
		> 6	1	0	NA	NA	0.34	-0.59	1.27
	Frequency (per week)	1	7	6	0.87	0%	0.19	-0.03	0.4
		2	2	1	0.49	0%	0.55	0.02	1.08
		3	2	1	0.38	0%	2.69	2.07	3.31
		4	1	0	NA	NA	0.34	-0.59	1.27
	Duration (minutes)	≤ 30	6	5	0.00	0%	0.95	0.06	1.84
		30–60	3	2	0.68	91%	0.47	0.05	0.88
		> 60	1	0	NA	NA	0.61	-0.25	1.47

*N *Number of studies, *NA *Not available

#### Publication bias and sensitivity analysis

Funnel plots showed some asymmetry, suggesting the possibility of publication bias (Figures S3 and S4). Egger’s test further supported this finding, with p values < 0.05 for both outcomes, indicating potential publication bias and small-study effects. To assess the robustness of the results, we applied the trim-and-fill method. After adjusting for potentially missing studies, the pooled effect sizes for both anxiety and athletic performance were reduced, suggesting that publication bias may have influenced the magnitude of the observed effects (Figures S5 and S6). Sensitivity analyses were performed to evaluate the stability of the results. For competitive anxiety outcomes, analyses were repeated after excluding small-sample studies (Figure S7). For athletic performance outcomes, analyses were conducted under three scenarios: excluding small-sample studies, excluding non-randomized trials, and excluding both (Figures S8–S10; Table S7). Across all scenarios, the pooled effect estimates remained largely unchanged in both magnitude and direction, with no meaningful differences in statistical significance. These findings indicate that the results are robust and not unduly influenced by study size or design. Although publication bias may have affected the estimated effect sizes, the consistency of the findings across sensitivity analyses supports the overall stability of the results.

#### Quality of evidence

The certainty of the evidence was assessed using the GRADE approach (Table S8). The evidence for competitive anxiety was rated as low, largely due to study limitations and potential reporting bias. Similarly, the evidence for athletic performance was rated as low, primarily because of the inclusion of non-randomized trials and reporting bias.

## Discussion

This study aims to synthesize existing studies to explore the effects of psychological intervention on competitive anxiety and athletic performance in golfers, and to explore the effects of covariates such as athletes’ competitive level, intervention type, and intervention duration on the effects of psychological intervention through subgroup analysis. The results showed that the psychological intervention were associated with reductions in competitive anxiety and athletic performance in golfers compared to the control group. Exploratory subgroup analyses suggested that larger effect sizes were observed among male and amateur golfers. Interventions delivered for less than 30 min twice weekly over more than six weeks tended to be associated with larger reductions in competitive anxiety, whereas interventions lasting up to 30 min three times weekly for 3–6 weeks showed relatively larger improvements in athletic performance.

Psychological interventions were associated with reductions in competitive anxiety and improvements in athletic performance among golfers. These findings align with those of Ong et al. (2021) [43] and Reinebo et al. (2024) [44], who reported small-to-moderate and large effect sizes, respectively, for psychological interventions on athletes’ competitive anxiety and performance. However, unlike their reviews, which synthesized evidence across multiple sports, the present study focuses specifically on golf. Given that golf is a closed-skill sport with relatively high levels of competitive anxiety, the impact of such anxiety may be particularly detrimental to performance [45]. Psychological interventions aim to regulate internal states by helping athletes manage or eliminate negative thoughts, emotions, and feelings, which may facilitate psychological readiness for competition [46, 47]. Moreover, these interventions may also enhance performance by promoting or sustaining functional athletic behaviors (FAB) [48].

The present study suggests that psychological interventions may be associated with relatively larger effect sizes in male golfers. This contrasts with the findings of Ong et al. (2021), who reported that psychological interventions were most effective in alleviating competitive anxiety among female athletes [43]. Nonetheless, other studies support our results, indicating that women tend to appraise stressors more severely, exhibit higher levels of anxiety and threat perception, and consequently engage in a greater number of coping strategies, which may diminish the effectiveness of interventions [49, 50]. However, these findings should be interpreted cautiously, as female participants were underrepresented in the included studies and only one female-only study contributed to the athletic performance analyses, limiting the ability to draw firm conclusions regarding sex differences. Moreover, our findings revealed that psychological interventions were particularly effective for amateur golfers, with comparable benefits observed among novice players. This pattern may reflect differences in competitive experience. Amateur and novice golfers often report higher levels of anxiety than elite players, largely due to their limited exposure to competitive settings, which can negatively affect both physical execution and mental focus [51]. At the same time, these golfers may rely more heavily on psychological skills, making them more responsive to structured psychological interventions and potentially amplifying their effects [51, 52]. It should be noted that studies involving elite golfers were not included due to the predefined inclusion criteria. Nevertheless, prior research has also suggested potential benefits of psychological interventions in this population. Elite athletes have been shown to employ psychological skills more extensively than less skilled counterparts, relying more on general psychological strategies, exhibiting more advanced psychomotor skills, and experiencing lower levels of competitive anxiety [22]. Furthermore, psychological interventions have been associated with enhanced athletic performance in elite golfers [53].

Mindfulness-based interventions tended to be associated with larger reductions in competitive anxiety, whereas imagery interventions tended to show relatively greater improvements in athletic performance; however, these findings were based on a limited number of studies and should be interpreted cautiously, and no conclusions regarding the relative superiority of intervention types can be drawn. This pattern may be consistent with previous studies by Perry et al. (2017) and Thompson et al. (2011), which reported reductions in competitive anxiety, particularly cognitive anxiety, following mindfulness-based training [54, 55]. Mindfulness may help athletes attend to present-moment experiences in an accepting and non-judgmental manner, rather than attempting to suppress or control negative internal states [56, 57]. Unlike traditional psychological skills training, which focuses on modifying maladaptive thoughts and emotions, mindfulness instead targets the athlete’s relationship with these experiences, which may help explain its potential utility in reducing competitive anxiety [44, 58, 59]. Relaxation techniques showed comparatively smaller effects in the present review, although previous studies have reported benefits for both cognitive and somatic anxiety [22, 60]. One possible explanation is that progressive muscle relaxation was the primary technique used, and limited supervision during practice may have reduced adherence and effectiveness.

Similarly, imagery interventions tended to be associated with relatively greater improvements in performance outcomes; however, the evidence base remains limited, and comparisons across intervention types should be interpreted with caution. Imagery may allow golfers to mentally rehearse key technical and tactical elements such as posture, grip, and shot execution, which could contribute to improved skill familiarity and arousal regulation, thereby potentially enhancing performance [26–28]. Goal-setting interventions also appeared to be associated with beneficial effects on both competitive focus and performance, consistent with findings from broader sport psychology literature [61, 62]. Since its development in the mid-1980s, goal-setting theory has generally suggested that specific, challenging, and measurable goals are associated with higher levels of performance compared with vague or easy goals [63, 64]. Although mindfulness–acceptance–commitment (MAC) interventions showed less pronounced effects in this analysis, previous research has reported beneficial effects on both competitive anxiety and performance [65–67]. MAC emphasizes psychological flexibility by encouraging athletes to accept internal experiences without judgment while committing to value-consistent actions [57]. More recently, mindfulness-based approaches such as the Mindfulness Sport Enhancement Program (MSPE) [68] and Mindfulness, Acceptance, and Self-Compassion approaches (MASCS) [69] have received increasing attention. Future research should further examine the application of these approaches in golf contexts. Overall, differences across intervention types should be interpreted cautiously and considered exploratory, and should not be taken as evidence of the superiority of any specific intervention approach.

The effectiveness of psychological interventions on competitive anxiety tended to increase with longer intervention duration, with larger effects observed in programs lasting more than six weeks. This aligns with findings by Wang et al. (2024), who reported that mindfulness interventions of more than seven weeks produced stronger effects than shorter programs [46]. Because competitive anxiety is a common challenge in sport, interventions that extend over time may provide athletes with sufficient opportunity to internalize and practice psychological skills, such as imagery and relaxation, which are also part of routine golf training. For athletic performance, interventions lasting three to six weeks appeared to be most effective. Both shorter and longer intervention periods were associated with reduced benefits. One possible explanation is that athletes may gain the greatest advantage when interventions coincide with the pre-competition period, allowing them to reach and sustain an optimal psychological state in the weeks leading up to competition [70]. In terms of frequency and duration, moderate intervention programs—typically two to three sessions per week lasting less than 30 min—appeared to be associated with favorable outcomes in both competitive anxiety and athletic performance. Deviations from this pattern, either by increasing or decreasing frequency or by extending session length, were linked to diminished outcomes. This aligns with prior research suggesting that repeated efforts to control or suppress internal states may lead to rebound effects, depleting limited cognitive resources and reducing adaptability to situational demands [46, 71]. Such psychological rigidity can interfere with meaningful behavior [72] and negatively affect both mental health and athletic performance [73]. Finally, research has shown that athletes who regularly use psychological skills experience lower competitive anxiety during competition, regardless of their skill level [17, 74, 75]. These findings may indicate that intervention frequency plays an important role in shaping outcomes; however, given the exploratory nature of the subgroup analyses and the absence of significant moderators in the meta-regression analyses, this interpretation should be considered preliminary and requires confirmation in future studies.

### Practical implications

These findings suggest that psychological interventions may have potential value for managing competitive anxiety and supporting athletic performance in golfers. They provide preliminary evidence that may inform athletes, coaches, and practitioners when considering the integration of psychological strategies into golf training programs. Given the prevalence of competitive anxiety and its potential impact on performance, incorporating psychological skills training into regular practice may be beneficial. Exploratory subgroup analyses suggested that interventions delivered two to three times per week and lasting up to 30 min per session tended to show relatively larger effects. Although these findings may serve as a preliminary reference for designing psychological intervention programs in golf, they are based on limited evidence and require further confirmation in future studies.

### Limitations and future directions

This study has several limitations. First, meta-regression analyses of participant and intervention characteristics did not identify any significant moderators. The substantial heterogeneity observed for athletic performance outcomes may therefore be attributable to unmeasured factors, including differences in intervention adherence, practitioner expertise, and delivery settings. The limited number of studies, the presence of outlier effect sizes, and potential ceiling effects among higher-skilled participants may have further contributed to the observed heterogeneity. In addition, the included studies assessed different dimensions of golf performance, including putting performance, overall scoring performance, and short-game performance. Although these outcomes were synthesized as objective indicators of golf-specific performance, their conceptual differences may also have contributed to between-study variability. Accordingly, the pooled estimate for athletic performance should be interpreted with caution and confirmed in future studies using more homogeneous outcome measures and intervention protocols. Second, most included RCTs did not report blinding procedures, and among excluded RCTs, the primary reason for exclusion was insufficient data reporting. Adherence to CONSORT guidelines in future studies will enhance transparency and methodological rigor. Third, some subgroup analyses were based on a limited number of studies or lacked directly comparable data, particularly for elite and female athletes. This imbalance reflects the underlying composition of the available evidence and may limit the robustness and generalizability of findings for these subgroups, while results for male and amateur athletes are comparatively better supported. These results should therefore be interpreted with caution. In addition, no statistically significant moderators were identified in the meta-regression analyses. Therefore, the observed subgroup differences should be considered exploratory and hypothesis-generating rather than definitive evidence of differential intervention effects, and should be interpreted with caution. Moreover, as the included studies primarily involved amateur and novice golfers, the findings are most applicable to these populations and cannot be readily generalized to elite athletes. Future research should prioritize more balanced representation across gender and competitive levels, as well as further examine the effectiveness of emerging interventions, such as mindfulness-based approaches, in golf contexts. Fourth, although sensitivity analyses indicated that the main findings were generally robust, funnel plot asymmetry, significant Egger’s tests, and trim-and-fill analyses suggested the presence of publication bias. Consequently, the pooled effect estimates may have been overestimated, and the observed benefits should be interpreted with caution. In addition, the inclusion of non-randomized studies may have further reduced the certainty of the evidence. Only English-language publications were included in this review, and relevant studies published in other languages may have been missed, introducing potential language bias and limiting the comprehensiveness of the evidence base. Future research should expand searches to include non-English literature and prioritize large, prospectively registered randomized controlled trials to reduce bias and improve the reliability of effect estimates. Fifth, none of the included studies assessed the directional interpretation of competitive anxiety, limiting a more nuanced understanding of how anxiety influences performance. Previous research has emphasized that anxiety symptoms may be perceived as either facilitative or debilitative, with important implications for performance outcomes [76, 77]. Furthermore, Jones et al. (2019) proposed a revised multidimensional model of competitive anxiety that refines the assessment of cognitive and somatic anxiety while incorporating a regulatory dimension related to perceived control [6]. Future research should incorporate validated measures of anxiety directionality, such as the Competitive Anxiety Direction Scale and the revised framework proposed by Jones et al. (2019), to distinguish between facilitative and debilitative interpretations of anxiety and to support more targeted intervention strategies [43]. Finally, advances in virtual reality (VR) and biofeedback have introduced innovative training approaches, including VR-based eye-tracking feedback, neurofeedback combined with VR, and VR interventions that simulate competitive situations [78–80]. These methods show promise for improving competitive anxiety and performance, and future studies should explore their feasibility and effectiveness in golf training. Taken together, these limitations should be considered when interpreting the findings of the present review. For transparency and to facilitate interpretation, a summary of the principal methodological limitations and potential sources of bias is provided in Table S9.

## Conclusion

This study synthesized the available evidence on the effects of psychological interventions on competitive anxiety and athletic performance in golfers. Psychological interventions were associated with small reductions in competitive anxiety and moderate improvements in athletic performance. Exploratory subgroup analyses suggested that larger effects may be observed among male and amateur golfers and in studies using interventions delivered in sessions of up to 30 min, two to three times per week. However, given the low certainty of the evidence, evidence of publication bias, substantial heterogeneity in athletic performance outcomes, and the exploratory nature of the subgroup analyses, these findings should be interpreted cautiously and warrant confirmation in future high-quality studies. Psychological interventions may nevertheless have potential value as part of golf training programs. Future research should prioritize high-quality randomized controlled trials with more balanced representation across sex and competitive level, further examine specific dimensions of competitive anxiety, and evaluate emerging approaches, including mindfulness-based programs and technology-assisted interventions such as virtual reality and biofeedback.

## Supplementary Information


Supplementary Material 1.



Supplementary Material 2.


## Data Availability

All data generated or analysed during this study are included in this published article [and its supplementary information files].

## Abbreviations

CBT: Cognitive Behavioral Therapy
CI: Confidence Interval
GRADE: Grading of Recommendations Assessment, Development, and Evaluation
MAC: Mindfulness–Acceptance–Commitment
PRISMA: Preferred Reporting Items for Systematic Reviews and Meta-Analyses
PROSPERO: International Prospective Register of Systematic Reviews
RCT: Randomized Controlled Trial
SMD: Standardized Mean Difference
VR: Virtual Reality
