# Supplementary material for: Effects of psychological interventions on competitive anxiety and athletic performance in golfers: a systematic review and meta-analysis
Source: BMC Sports Sci Med Rehabil. 2026 Aug 27;18:388. doi: 10.1186/s13102-026-01888-2 (PMC13536777; doi:10.1186/s13102-026-01888-2)
Supplement: Supplementary file 2 [file 13102_2026_1888_MOESM2_ESM.doc]

**Effects of psychological interventions on competitive anxiety and athletic performance in golfers: a systematic review and meta-analysis**

**Supplementary Material**

| **Supplemental material** | **Page** |
| --- | --- |
| **Supplementary Table 1: Search strategy** | 1 |
| **Supplementary Table 2: Excluded studies and reasons for exclusion** | 2-4 |
| **Supplementary Table 3: Study characteristics** | 5-8 |
| **Supplementary Table 4. Risk of bias assessment (ROB 2)** | 9 |
| **Supplementary Table 5. Risk of bias assessment (ROBINS-I V2)** | 9 |
| **Supplementary Table 6: Meta regression of athletic performance outcomes** | 10 |
| **Supplementary Figure 1: Subgroup analysis of competitive anxiety outcomes based on intervention type.** | 10 |
| **Supplementary Figure 2. Subgroup analysis of athletic performance outcomes based on intervention type.** | 11 |
| **Supplementary Figure 3: Funnel plot of competitive anxiety outcomes.** | 12 |
| **Supplementary Figure 4: Funnel plot of athletic performance outcomes.** | 12 |
| **Supplementary Figure 5. The trim-and-fill method for competitive anxiety outcomes.** | 13 |
| **Supplementary Figure 6. The trim-and-fill method for athletic performance outcomes.** | 13 |
| **Supplementary Table 7. Sensitive analysis excludes studies** | 14 |
| **Supplementary Figure 7. Sensitivity analysis of competitive anxiety outcomes excluding small sample studies.** | 14 |
| **Supplementary Figure 8. Sensitivity analysis of athletic performance outcomes excluding small sample studies.** | 15 |
| **Supplementary Figure 9. Sensitivity analysis of athletic performance outcomes excluding Non-randomized controlled study.** | 15 |
| **Supplementary Figure 10. Sensitivity analysis of athletic performance outcomes excluding small sample and non-randomized controlled studies.** | 16 |
| **Supplementary Table 8. Quality of Evidence** | 17 |
| **Supplementary Table 9. Summary of key limitations of the included evidence** | 18 |

**Supplementary Table 1. Search strategy**

| **Wos** | TS=("psychological intervention*" OR "mental skill*" OR "mental training" OR psychological*)  AND  TS=("competitive anxiety" OR anxiety* OR worry* OR "athletic performance" OR "sports performance" OR performance*)  AND  TS=(golfer* OR golf*) |
| --- | --- |
| **Pubmed** | ("psychological intervention"[Title/Abstract] OR "mental skill*"[Title/Abstract] OR "mental training"[Title/Abstract] OR psychological*[Title/Abstract])  AND  ("competitive anxiety"[Title/Abstract] OR anxiety[Title/Abstract] OR worry[Title/Abstract] OR "athletic performance"[Title/Abstract] OR "sports performance"[Title/Abstract] OR performance[Title/Abstract])  AND  (golfer*[Title/Abstract] OR golf*[Title/Abstract]) |
| **Embase** | ('psychological intervention':ab,ti OR 'mental skill*':ab,ti OR 'mental training':ab,ti OR 'psychological':ab,ti)  AND  ('competitive anxiety':ab,ti OR 'anxiety':ab,ti OR 'worry':ab,ti OR 'athletic performance':ab,ti OR 'sports performance':ab,ti OR 'performance':ab,ti)  AND  ('golfer':ab,ti OR 'golf':ab,ti) |
| **Cochrane** | ("psychological intervention*" OR "mental skill*" OR "mental training" OR psychological*):ti,ab,kw  AND  ("competitive anxiety" OR anxiety OR worry OR "athletic performance" OR "sports performance" OR performance):ti,ab,kw  AND  (golfer* OR golf*):ti,ab,kw |
| **Scopus** | TITLE-ABS-KEY(("psychological intervention*" OR "mental skill*" OR "mental training" OR psychological*) AND ("competitive anxiety*" OR anxiety* OR worry* OR "athletic performance*" OR "sports performance*" OR performance*) AND (golfer* OR golf*)) |
| **Google**  **Scholar** | ("psychological intervention" OR "mental skill" OR "mental training" OR psychological) AND ("competitive anxiety" OR anxiety OR worry OR "athletic performance" OR "sports performance" OR performance) AND (golfer OR golf) |

**Supplementary Table 2. Excluded studies and reasons for exclusion**

| **Study** | **Reasons for Exclusion** |
| --- | --- |
| Balk et al. (2013) [1] | No data |
| Bell and Hardy (2009) [2] | No control |
| Brouziyne and Molinaro (2005) [3] | No control |
| Chen et al. (2022) [4] | No data |
| He et al. (2024) [5] | Not control |
| Kao et al. (2013) [6] | No control |
| Kaufman et al. (2009) [7] | No data |
| Kim et al. (2017) [8] | No data |
| Larsson et al. (1988) [9] | No data |
| Moore et al. (2013) [10] | No data |
| Nicholls et al. (2005) [11] | No control |
| Quinton et al. (2016) [12] | No control |
| Smith et al. (2004) [13] | No data |
| Turner et al. (2018) [14] | No control |
| Wang et al. (2020) [15] | No control |
| Wang et al. (2023) [16] | No data |
| Wu et al. (2024) [17] | No control |

[1] Balk YA, Adriaanse MA, De Ridder DT, Evers C. Coping under pressure: Employing emotion regulation strategies to enhance performance under pressure. J Sport Exerc Psychol. 2013; doi: 10.1123/jsep.35.4.408.
[2] Bell JJ, Hardy J. Effects of attentional focus on skilled performance in golf. J Appl Sport Psychol. 2009; doi: 10.1080/10413200902795323.
[3] Brouziyne M, Molinaro C. Mental imagery combined with physical practice of approach shots for golf beginners. Percept Mot Skills. 2005; doi: 10.2466/pms.101.1.203-21.
[4] Chen TT, Wang KP, Chang WH, Kao CW, Hung TM. Effects of the function-specific instruction approach to neurofeedback training on frontal midline theta waves and golf putting performance. Psychol Sport Exerc. 2022; doi: 10.1016/j.psychsport.2022.102211.
[5] He Q, Liu Y, Yang Y. The effect of quiet eye training on golf putting performance in pressure situation. Sci Rep. 2024; doi: 10.1038/s41598-024-55716-z
[6] Kao SC, Huang CJ, Hung TM. Neurofeedback training reduces frontal midline theta and improves putting performance in expert golfers. J Appl Sport Psychol. 2014; doi: 10.1080/10413200.2013.855682.
[7] Kaufman KA, Glass CR, Arnkoff DB. Evaluation of Mindful Sport Performance Enhancement (MSPE): A new approach to promote flow in athletes. J Clin Sport Psychol. 2009; doi: 10.1123/jcsp.3.4.334.
[8] Kim T, Frank C, Schack T. A systematic investigation of the effect of action observation training and motor imagery training on the development of mental representation structure and skill performance. Front Human Neurosci. 2017; doi: 10.3389/fpsyg.2023.1273186.
[9] Larsson G, Cook-Steffy C, Starrin B. Time and cost efficient stress inoculation training for athletes: A study of junior golfers. Scand J Sport Sci. 1988;10:23-28.
[10] Moore LJ, Vine SJ, Freeman P, Wilson MR. Quiet eye training promotes challenge appraisals and aids performance under elevated anxiety. Int J Sport Exerc Psychol. 2013; doi: 10.1080/1612197X.2013.773688.
[11] Polman R. The Effects of Individualized Imagery Interventions on Golf Performance and Flow States. Athl Insight, 2005;7(1), 43-66.
[12] Quinton ML, Cumming J, Allsop J, Gray R, Williams SE. Imagery meaning and content in golf: Effects on performance, anxiety, and confidence. Int J Sport Exerc Psychol. 2018; doi: 10.1080/1612197X.2016.1242150.
[13] Smith D, Holmes P. The effect of imagery modality on golf putting performance. J Sport Exerc Psychol. 2004; doi: 10.1123/jsep.26.3.385.
[14] Turner MJ, Kirkham L, Wood AG. Teeing up for success: The effects of rational and irrational self-talk on the putting performance of amateur golfers. Psychol Sport Exerc. 2018; doi: 10.1016/j.psychsport.2018.06.012.
[15] Wang HT, Tai HL, Yang CC, Chen YS. Acute effects of self-selected music intervention on golf performance and anxiety level in collegiate golfers: A crossover study. Int J Envir Res Public Health. 2020; doi: 10.3390/ijerph17207478.
[16] Wang KP, Cheng MY, Elbanna H, Schack T. A new EEG neurofeedback training approach in sports: the effects function-specific instruction of Mu rhythm and visuomotor skill performance. Front Psychol. 2023; doi: 10.3389/fpsyg.2023.1273186.
[17] Wu JH, Tu YC, Chang CY, Chueh TY, Gentili RJ, Hatfield BD, Hung TM. A single session of sensorimotor rhythm neurofeedback enhances long-game performance in professional golfers. Biol Psychol. 2024; doi: 10.1016/j.biopsycho.2024.108844.

**Supplementary Table 3. Study characteristics.**

| **Author** | **Country** | **Athlete level** | **Female percentage** | **Age**  **(mean)** | **Samp size** | **Intervention type** | **Length**  **(week)** | **Frequency**  **(a week)** | **Duration**  **(min)** | **Outcome measurement** | **Study design** |
| --- | --- | --- | --- | --- | --- | --- | --- | --- | --- | --- | --- |
| Bouchard (2000) [1] | USA | Amateur | 0.00% | 22.25 | 40 | CBT | 1 | 1 | 60 | MCSAI-2\Short Game Performance | RCT |
| DeSchryver (1992) [2] | USA | Amateur | 51.72% | NA | 56 | Relaxation | 5 | 1 | 30 | The Strokes of 18 Holes | RCT |
| Grobbelaar et al. (2018) [3] | South Africa | Amateur | 0.00% | 20.37 | 14 | PST | 5 | 1 | 60 | CSAI-2 | RCT |
| Ismail (2016) [4] | Malaysia | Amateur | 0.00% | 20.83 | 42 | Imagery | 6 | 3 | 10 | CSAI-2R\  Putting Performance | RCT |
| Kingston and Hardy (1997) [5] | UK | Amateur | 0.00% | 44.15 | 37 | Goal Setting | 54 | 2 | 20 | CSAI-2 | RCT |
| Kornblath (1985) [6] | USA | Amateur | 22.50% | 56.57 | 38 | PST | 4 | 2 | NA | The Strokes of 18 Holes | NRCT |
| Lin et al. (2022) [7] | China | Novice | 51.11% | 20.18 | 45 | Imagery | 6 | 3 | 20 | Putting Performance | RCT |
| Ortiz and Grange (2006) [8] | USA | Amateur | 100.00% | 33 | 18 | Relaxation | 12 | 4 | 20 | Putting Performance | RCT |
| Perry et al. (2017) [9] | USA | Novice | 49.23% | 18.73 | 65 | Mindfulness | 1 | 1 | 30 | STAI\Putting Performance | RCT |
| Plemons (2015) [10] | USA | Amateur | 0.00% | 24.37 | 19 | MAC | 5 | 2 | 60 | SAS-2\Putting Performance | RCT |

**Supplementary Table 3. Study characteristics.**

| **Author** | **Country** | **Athlete level** | **Female percentage** | **Age**  **(mean)** | **Samp size** | **Intervention type** | **Length**  **(week)** | **Frequency**  **(a week)** | **Duration**  **(min)** | **Outcome measurement** | **Study design** |
| --- | --- | --- | --- | --- | --- | --- | --- | --- | --- | --- | --- |
| Ramsey et al. (2011) [11] | UK | Novice | 52.00% | 21 | 75 | Imagery | 1 | 1 | 30 | Putting Performance | RCT |
| Shaffery (1996) [12] | USA | Amateur | 30.56% | NA | 36 | IE/CBT | 4 | 1 | 45 | CSAI-2\The Strokes  of 18 Holes | RCT |
| Shivetts (2006) [13] | USA | Amateur | 0.00% | 18-24 | 43 | Goal Setting | 4 | NA | NA | Putting Performance | RCT |
| Short et al. (2002) [14] | USA | Amateur | 43.37% | 21.44 | 52 | Imagery | 1 | 1 | NA | Putting Performance | NRCT |
| Wager (1983) [15] | USA | Amateur | 0.00% | 14-18 | 25 | Imagery/  Relaxation | 4 | 1 | 90 | SCAT\Putting Performance | RCT |

CBT: Cognitive Behavioral Therapy; CSAI-2: The Competitive State Anxiety Inventorv-2; CSAI-2R: The Competitive State Anxiety Inventorv-2 Revised; IE: Imaginal Exposure; MAC: Mindfulness-Acceptance-Commitment; MCSAI-2: The Modified Competitive State Anxiety Inventorv-2; NA: Not Reported; NRCT: Non-randomized Controlled Trial; PST: Psychological Skills Training; RCT: Randomized Controlled Trial; SAS-2: The Sports Anxiety Scale-2; SCAT: Sport Competition Anxiety Test; STAI: The State-Trait Anxiety Inventory Scale; UK: United Kingdom; USA: United States of America.

[1] **Bouchard LJ. The effects of a cognitive and somatic awareness intervention on competitive golf performance and emotion regulation. [PhD thesis]. Florida: University of Florida; 2000.**
[2] **DeSchryver MA.  Effects of chaining-mastery instruction, anxiety management training, and bibliotherapy on golf performance. [PhD thesis].  New York: Hofstra University;1992.**
[3] **Grobbelaar HW, Duthie KR, Fanton ER. Effect of a group multimodal anxiety management programme on competitive state anxiety and self-confidence of amateur golfers. South African Journal for Research in Sport, Physical Education and Recreation. 2018;40(2):69-80.**
[4] **Ismail M. Effectiveness of “pim” training on putting performance and pre-competitive anxiety of the golfers. Int J Golf Sci. 2016; doi: 10.1123/ijgs.2014-0013.**
[5] **Kingston KM, Hardy L. Effects of different types of goals on processes that support performance. Sport Psychol. 1997; doi: 10.1123/tsp.11.3.277.**
[6] **Kornblath R. A cognitive-behavioral Approach to stress management with golfers. [PhD thesis]. Los Angeles (CA): California School of Professional Psychology - Los Angeles; 1985.**
[7] Lin CH, Lu FJ, Gill DL, Huang KS, Wu SC, Chiu YH. Combinations of action observation and motor imagery on golf putting’s performance. PeerJ. 2022; **doi: 10.7717/peerj.13432.**
[8] **Ortiz J, Grange LL. Efficacy of relaxation techniques in increasing sport performance in women golfers. Sport J. 2016;24:1-7.**
[9] Perry JE, Ross M, Weinstock J, Weaver T. Efficacy of a brief mindfulness intervention to prevent athletic task performance deterioration: A randomized controlled trial. Sport Psychol. 2017; **doi: 10.1123/tsp.2016-0130.**
[10] **Plemmons MG. Evaluation of the effectiveness of the Mindfulness-Acceptance-Commitment (MAC) approach in recreational golfers. [Master’s thesis]. Boone: Appalachian State University; 2015.**
[11] **Ramsey R, Cumming J, Edwards MG. Exploring a modified conceptualization of imagery direction and golf putting performance. Int J Sport Exerc Psychol. 2008; doi: 10.1080/1612197X.2008.9671862.**
[12] **Shaffery, JP. Effects of Imaginal Flooding on Golf Putting Performance. [PhD thesis]. New York: Hofstra University; 1996.**
[13] **Shivetts, MS. Effects of an Individual Goal-Setting Intervention on Goal Orientation, Self-Confidence, and Riving Accuracy in Average Golfers: A Quantitative. [Master’s thesis]. Statesboro: Georgia Southern University; 2006.**
[14] **Short SE, Bruggeman JM, Engel SG, Marback TL, Wang LJ, Willadsen A, Short MW. The effect of imagery function and imagery direction on self-efficacy and performance on a golf-putting task. Sport Psychol. 2002; doi: 10.1123/tsp.16.1.48.**
[15] **Wager DB. Sports anxiety reduction techniques to improve golf performance. [PhD thesis].  New York: Hofstra University; 1983.**

**Supplementary Table 4. Risk of Bias Assessment Based on the PEDro Scale**

| **Study** | **Randomization process** | **Deviations from intended interventions** | **Missing outcome data** | **Measurement of the outcome** | **Selection of the reported result** | **Overall Bias** |
| --- | --- | --- | --- | --- | --- | --- |
| Bouchard (2000) | L | L | L | L | L | L |
| DeSchryver (1992) | S | L | L | L | L | S |
| Ortiz and Grange (2006) | L | L | L | L | L | L |
| Grobbelaar et al.(2018) | L | L | L | L | L | L |
| Ismail (2016) | L | L | L | L | L | L |
| Kingston and Hardy(1997) | S | L | L | L | L | S |
| Lin et al. (2022) | L | S | L | L | L | S |
| Perry et al.(2017) | L | L | L | L | L | L |
| Plemons (2015) | L | L | L | L | L | L |
| Ramsey et al.(2011) | L | S | L | L | L | L |
| Shaffery (1996) | L | L | L | L | L | L |
| Short et al. (2002) | L | L | L | L | L | L |
| Wager (1983) | L | L | S | L | L | S |

**Supplementary Table 5. Risk of Bias Assessment Based on the PEDro Scale**

| **Study** | **Bias due to confounding** | **Bias in classification of interventions** | **Bias in selection of participants into the study** | **Bias due to missing data** | **Bias in measurement of the outcome** | **Bias in selection of the reported result** | **Overall risk of bias** |
| --- | --- | --- | --- | --- | --- | --- | --- |
| Kornblath (1985) | M | L | M | L | L | L | M |
| Shivetts (2006) | M | L | M | L | L | L | M |

**Supplementary Table 6. Meta regression of athletic performance outcomes**

| **Covariates** | **Coefficient** | **Standard error** | **z** | **P>\|z\|** | **95% CI** | |
| --- | --- | --- | --- | --- | --- | --- |
| Year | -0.14 | 0.13 | -1.08 | 0.28 | -0.38 | 0.11 |
| Country | 0.62 | 0.60 | 1.03 | 0.30 | -0.56 | 1.80 |
| Athlete Level | 0.62 | 1.42 | 0.43 | 0.67 | -2.18 | 3.41 |
| Geder | 0.03 | 0.56 | 0.05 | 0.96 | -1.07 | 1.13 |
| Age | -0.04 | 0.02 | -1.49 | 0.14 | -0.09 | 0.01 |
| Samp Size | -0.06 | 0.08 | -0.74 | 0.46 | -0.21 | 0.10 |
| Type | 0.20 | 0.27 | 0.73 | 0.46 | -0.33 | 0.73 |
| Length | -0.29 | 0.24 | -1.21 | 0.23 | -0.76 | 0.18 |
| Frequency | 0.63 | 1.15 | 0.55 | 0.59 | -1.63 | 2.89 |
| Duration | -0.02 | 0.02 | -1.30 | 0.19 | -0.05 | 0.01 |

CI: confidence interval

**Supplementary Figure 1. Subgroup analysis of competitive anxiety outcomes based on intervention type. CBT: Cognitive Behavioral Therapy; GS: Goal Setting; IE:** Imaginal Exposure; **IM: Imagery; MF: Mindfulness; MAC: Mindfulness-Acceptance-Commitment; PST: Psychological Skills Training; RE: Relaxation.**

**Supplementary Figure 2. Subgroup analysis of athletic performance outcomes based on intervention type. CBT: Cognitive Behavioral Therapy; GS: Goal Setting; IE:** Imaginal Exposure; **IM: Imagery; MF: Mindfulness; MAC: Mindfulness-Acceptance-Commitment; PST: Psychological Skills Training; RE: Relaxation.**

**Supplementary Figure 3. Funnel plot of competitive anxiety outcomes.**

**Supplementary Figure 4. Funnel plot of athletic performance outcomes.**

**Supplementary Figure 5. The trim-and-fill method for competitive anxiety outcomes.**

**Supplementary Figure 6. The trim-and-fill method for athletic performance outcomes.**

**Supplementary Table 7. Sensitive analysis excludes studies**

| Small sample studies | Ortiz and Grange (2006) |
| --- | --- |
|  | **Plemons (2015)** |
| Non-randomized controlled studies | **Kornblath (1985)** |
|  | **Short et al. (2002)** |

[1] **Ortiz J, Grange LL. Efficacy of relaxation techniques in increasing sport performance in women golfers. Sport J. 2016;24:1-7.**
[2] Plemmons MG. Evaluation of the effectiveness of the Mindfulness-Acceptance-Commitment (MAC) approach in recreational golfers. [Master’s thesis]. Boone: Appalachian State University; 2015.
[3] Kornblath R. A cognitive-behavioral Approach to stress management with golfers. [PhD thesis]. Los Angeles (CA): California School of Professional Psychology - Los Angeles; 1985.
[4] Short SE, Bruggeman JM, Engel SG, Marback TL, Wang LJ, Willadsen A, Short MW. The effect of imagery function and imagery direction on self-efficacy and performance on a golf-putting task. Sport Psychol. 2002; doi: 10.1123/tsp.16.1.48.

**Supplementary Figure 7. Sensitivity analysis of competitive anxiety outcomes excluding small sample studies.**

**Supplementary Figure 8. Sensitivity analysis of athletic performance outcomes excluding small sample studies.**

**Supplementary Figure 9. Sensitivity analysis of athletic performance outcomes excluding Non-randomized controlled study.**

**Supplementary Figure 10. Sensitivity analysis of athletic performance outcomes excluding small sample and non-randomized controlled studies.**

**Supplementary Table 8. Quality of Evidence**

| **Comparison**  **(Outcome)** | **SMD**  **(95% CI)** | **Number of Studies** | **Design** | **Study**  **Limitations** | **Indirectness** | **Inconsistency** | **Imprecision** | **Publication Bias** | **Quality of Evidence** |
| --- | --- | --- | --- | --- | --- | --- | --- | --- | --- |
| PI vs Control  (C**ompetitive Anxiety**) | -0.29 (-0.57 to -0.02) | 8 | RCT | Serious^1^ | Little or no | Little or no | Little or no | Serious | ⊕⊕O O  Low |
| PI vs Control  (A**thletic Performance**) | 0.70 (0.28 to 1.12) | 13 | RCT and  NRCT | Serious^2^ | Little or no | Little or no | Little or no | Serious | ⊕⊕O O  Low |

^1^ Most of the included RCTs did not report blinding, and the risk of bias was unknown

^2^ Non-randomised controlled trials were included

CI: confidence interval; PI: Psychological Intervention; SMD: standardized mean difference.

**Supplementary Table 9. Summary of key limitations of the included evidence**

| **Limitation** | **Description** |
| --- | --- |
| Lack of blinding | Several studies did not report participant, assessor, or allocation blinding |
| Limited follow-up | Most studies assessed only immediate post-intervention outcomes |
| Underrepresentation of female golfers | Female participants accounted for only 26.7% of the total sample |
| Absence of elite golfers | Most participants were amateur or novice golfers |
| Small sample sizes | Several studies included fewer than 20 participants |
| Substantial heterogeneity in performance outcomes | High between-study heterogeneity (I² = 80.3%) |
